# 异基因造血干细胞移植后复发的免疫逃逸机制及治疗策略

**DOI:** 10.3760/cma.j.issn.0253-2727.2021.10.014

**Published:** 2021-10

**Authors:** 恒薇 吴, 妍敏 赵, 河 黄

**Affiliations:** 浙江大学附属第一医院骨髓移植中心，浙江大学血液学研究所，杭州 310006 Bone Marrow Transplantation Center, The First Affiliated Hospital, Zhejiang University; Institute of Hematology, Zhejiang University, Hangzhou 310006, China

异基因造血干细胞移植（allo-HSCT）是血液系统恶性疾病有效的治疗手段之一。感染和急性移植物抗宿主病（aGVHD）是allo-HSCT后早期死亡的主要原因，而在allo-HSCT 100 d后，白血病复发则是导致死亡的首要因素[1]。移植后高复发率和不良预后在过去几十年中没有得到明显改善[2]。尽管复发可能有许多因素在起作用，包括白血病细胞对预处理方案不敏感等，但在更多情况下是由于白血病细胞挣脱了供者T细胞的免疫监视，即发生了“免疫逃逸”[3]。因此，深入了解移植后复发的免疫生物学机制是选择治疗策略的基础。

一、allo-HSCT后白血病复发的机制

（一）HLA丢失与下调

在allo-HSCT后，白血病细胞的清除首先依赖于供者T细胞和自然杀伤细胞（NK）对它们的识别。T细胞以自身人类白细胞抗原（HLA）分子提呈的免疫原性肽以及非自身HLA的结构表位为识别目标，因此移植后的治疗效果取决于供者T细胞识别患者白血病上的肿瘤特异性抗原（TSA）、次要组织相容性抗原（mHAg）和错配HLA的能力[4]。而NK细胞则在白血病细胞上HLA Ⅰ类分子缺失或下调的情况下被激活[5]。

HLA Ⅰ类分子在所有有核细胞表面表达，主要向CD8^+^细胞毒性T细胞（CTL）的T细胞受体（TCR）呈递来自细胞内的肽链。因为白血病细胞特异性抗原通常是胞内蛋白，因此HLA Ⅰ类抗原对于CTL识别和杀伤肿瘤细胞至关重要。此外，NK细胞上的激活和抑制性杀伤细胞免疫球蛋白样受体（KIR）与一些CD8^+^T细胞亚群上可以识别并结合HLA Ⅰ类的B、C分子提呈的抗原[6]。CTL的TCR与肿瘤多肽-HLA复合物结合后被激活，而后CTL在肿瘤部位聚集并释放效应因子攻击肿瘤细胞。CTL主要通过两种途径对肿瘤细胞发挥特异性杀伤作用：①直接释放穿孔素、颗粒酶等细胞毒性物质；②激活死亡受体途径，如T细胞表面表达的Fas配体（FasL）与肿瘤细胞表面的Fas结合，启动凋亡相关信号导致肿瘤细胞凋亡。

与HLA Ⅰ类分子不同，HLA Ⅱ类分子的表达仅限于免疫细胞，主要向CD4^+^T细胞的TCR呈递来自细胞外蛋白的肽链，从而激活某些细胞毒效应[7]。

在移植背景下，HLA位点不相合会引起强烈的供者T细胞反应，特别是在单倍型造血干细胞移植（haplo-HSCT）中，患者和供者之间整个HLA单倍体的不匹配引起的同种异体反应更加剧烈[8]。haplo-HSCT后，患者细胞表面HLA Ⅰ类分子有一半是供者CTL不认识的，HLA Ⅱ类分子有一半对于供者CD4^+^辅助T细胞（Th）是陌生的，此时T细胞对不相合HLA介导的同种异体反应是移植物抗白血病（GVL）效应的主要组成部分；对于T细胞而言，同种异体反应的激活非常依赖TCR与异体的HLA的分子的相互作用[8]–[9]；对于发挥GVL效应的NK细胞而言，“missing self”假说认为未识别到相当水平的同种HLA Ⅰ类分子会使NK细胞发挥细胞毒作用[5]。因此，患者特异HLA缺失可能会导致白血病细胞逃过供者T细胞的免疫监控。

将移植前后患者白血病细胞与其供者T细胞共培养发现，供者T细胞可以攻击移植前的细胞，而对复发时患者特异性HLA丢失白血病细胞无反应；而来自HLA均不合的健康人T细胞，对移植前或复发时的患者白血病细胞均有相似反应[10]；以上实验表明，当白血病细胞失去患者特异性HLA时，供者T细胞会无视这群细胞，同时HLA不相合位点的数量与供者T细胞引起同种异体反应的激烈程度并非完全正相关。

除同种异体反应减弱之外，HLA缺失还会减少某些肿瘤抗原的提呈，进一步减弱CTL的杀伤效应[11]。肿瘤相关抗原需要与特定的HLA分子结合才可以诱导抗原特异性CTL产生。如细胞周期蛋白E1与E2都需要与HLA-A*02: 01结合才可诱导特异性CTL产生，故缺失该位点会使肿瘤抗原无法被提呈，阻碍特异性CTL产生[12]，继而减少CTL杀伤的总效能。KRAS G12D抗原特异性CTL对表达该抗原的结肠癌细胞进行杀伤。以往研究发现，一些结肠癌细胞由于HLA-C*08: 02缺失导致KRAS G12D抗原无法被提呈，因此该HLA基因座位丢失会直接导致免疫逃逸[13]。以上两个例子说明了某些HLA分子是肿瘤抗原提呈的必要条件。因此在allo-HSCT的背景下，患者特异性HLA位点丢失可能带来双重的复发机制：①CTL通过不相合HLA发挥同种异体免疫的GVL效应降低；②重要HLA位点缺失减少了白血病抗原提呈，不能诱导相应CTL产生，直接导致免疫逃逸。

在haplo-HSCT中还强调了NK细胞的GVL效应[14]。NK细胞杀伤肿瘤细胞依赖于激活性受体NCR、NKG2D、DNAM-1、NKG2C和抑制性受体KIR、NKG2A[15]。NK细胞在肿瘤细胞HLA Ⅰ类分子下调/丢失时被激活，这一现象在血液系统恶性肿瘤和实体瘤中都可以观察到[16]–[17]。HLA丢失的白血病细胞虽然阻碍了T细胞同种异体识别，但理论上NK细胞仍旧能行使同种异体反应：因为白血病细胞丢失的HLA等位基因通常也是供者KIR（抑制性受体）的配体，反过来可能会通过“配体丢失”机制增强NK细胞的杀伤能力[18]。

迄今为止，HLA丢失的分子驱动机制知之甚少，因为染色体断裂敏感性增加以及反复化疗诱导DNA损伤都有可能增加HLA丢失的发生率[19]，所以移植前接受过反复、大剂量化疗的患者可能更容易出现HLA丢失，并且移植后通过供者T细胞筛选，患者特异性HLA丢失的白血病细胞群最终会成为优势细胞亚群，但是需要强调，有的患者在初诊时就已出现HLA的丢失[20]，并且HLA的丢失可以仅发生于部分基因座位上[21]，因此无条件行HLA丢失检测的实验室会低估其发生率。

临床回顾性研究发现，haplo-HSCT后复发患者中HLA丢失占30％以上[22]–[23]，但不同的haplo-HSCT体系HLA丢失发生率是否相同，尚不能从现有的小规模病例报告中得出明确结论。对于不同的移植类型，HLA丢失复发在不全相合和全相合无关供者移植中都有发生，但发生率均低于haplo-HSCT[22]–[23]。haplo-HSCT后患者特异性HLA丢失的复发明显晚于“经典（classic）复发”（无HLA丢失的复发）且与移植前疾病活动状态相关[22]–[23]。移植前肿瘤负荷较大可能恶性细胞克隆的异质性也较高，更有可能携带HLA丢失的白血病细胞克隆。

除患者特异性HLA缺失外，HLA Ⅱ类分子表达下调也与allo-HSCT后复发相关。研究发现在移植后复发的患者中，40％都出现了HLA Ⅱ类分子（HLA-DR、-DQ和-DP）表达下调[24]–[25]。并且，复发时HLA Ⅱ类分子的表达水平较初发时低3～12倍[25]，因为HLA Ⅱ类分子和CD4^+^T细胞之间的相互作用是发挥GVL效应的必要条件，这种抗肿瘤免疫的机制给白血病细胞提供了一个可利用的弱点——通过减少HLA Ⅱ类分子的表达而实现免疫逃逸[26]。与HLA丢失不同，HLA Ⅱ类分子下调在各种移植类型中的发生率相似[24]–[25]，并且可能与移植物中较高含量的T细胞相关[24]。HLAⅡ类分子呈递mHAg的数量远远超过HLAⅠ类分子[27]–[28]，这可能说明HLA Ⅱ类分子下调相关的免疫压力并不是来自于不相合的HLA Ⅱ类分子，而是来自其呈递的肿瘤特异性抗原和mHAg[3]。一项研究结果发现HLA Ⅱ类分子下调与主要组织相容性复合体Ⅱ类反式激活因子（CIITA）的下调相关[29]。部分CIITA下调发生在表观遗传层面，如患者中观察到CIITA基因启动子甲基化或HLA Ⅱ类分子下调可被IFN-γ逆转等[24]–[25]。

HLA Ⅱ类分子的表达水平是判断恶性肿瘤预后的相关指标。在生发中心型（GCB型）弥漫性大B细胞淋巴瘤中，HLA Ⅱ类分子表达阴性与不良预后相关[30]–[31]。在一项程序性死亡受体1（PD-1）单抗治疗复发/难治经典霍奇金淋巴瘤的研究中，除了细胞程序性死亡-配体1（PD-L1）阳性表达外，HLA II类分子高表达的患者也能获得较高的反应率[32]。

（二）T细胞抑制性配体上调

“T细胞耗竭”概念的提出是用于描述在长期感染病毒的小鼠中持续存在但效应明显减弱的CD8^+^ T细胞的功能状态[33]。在慢性感染的背景下，持续性抗原刺激会导致CD8^+^T细胞效应功能丧失，即T细胞失去了产生白细胞介素2（IL-2）、肿瘤坏死因子-α（TNF-α）等细胞因子的能力，进而失去了增殖和细胞毒性杀伤的能力；同时还伴随T细胞表面抑制性受体的高表达，这一过程称为T细胞耗竭，是许多慢性感染和恶性肿瘤的共同特征[34]。PD-1是活化的T淋巴细胞所表达的抑制性共刺激受体，参与T细胞耗竭并起主要作用。当PD-1与抑制性配体PD-L1（B7-H1）、PD-L2（B7-DC）结合时，抑制性信号被激活[35]。PD-1在各种激活后的造血细胞中表达，如T细胞、B细胞、NK细胞、树突状细胞等；PD-L2的表达主要局限于抗原呈递细胞（APC），如B细胞、巨噬细胞等；而PD-L1在组织中广泛表达，且暴露于γ干扰素（IFN-γ）将进一步诱导其表达[35]。PD-1的功能在T细胞上研究得最为深入，其作为T细胞活化后的“刹车”，负调控T细胞效应来限制其过度活化，从而保护靶细胞免受T细胞过度地攻击。

在移植背景下，供者T细胞面对患者的异体抗原时常具有高反应性即高杀伤效能，但正是这种抗原持续存在的环境不可避免地提高了T细胞耗竭的可能性。一些研究发现，在治疗过程中，尤其是在allo-HSCT后，白血病细胞会增加抑制性T细胞分子或T细胞耗竭相关分子的表达[24]并会出现以产生细胞因子能力下降为特征的T细胞表型（如TNF-α、IL-2和IFN-γ产生减少）[36]。对比正常供者，接受allo-HSCT后复发患者CD4^+^和CD8^+^中心记忆T细胞和效应记忆T细胞表达更高的PD-1和CD57，这提示这些患者拥有更高的终末期/效应T细胞亚群[37]。并且，在移植后复发的患者中，抑制性受体的共表达会增加（约一半以上CD8^+^T细胞表达≥3种抑制性受体）[37]，这提示了T细胞功能严重受损以及慢性病毒感染状态。相反，在接受化疗的患者中，CD4^+^T细胞不论是单一抑制性受体表达水平或多种抑制性受体共表达量都与健康人相当；而对比CD8^+^T细胞，化疗后患者不论是单个抑制性受体或者其共表达均增加[38]。

有研究发现，在接受HLA全相合移植的患者中，外周血T细胞上PD-1的表达在移植后复发和非复发患者均有升高，这表明PD-1并不是allo-HSCT复发患者T细胞耗竭的唯一主要标志[39]。在急性髓系白血病（AML）患者中发现：PD-1高表达、T细胞免疫球蛋白黏蛋白分子-3（TIM-3）阳性T细胞与移植后复发密切相关，且这种T细胞产生的炎性细胞因子较少[40]。此外，CTL表面PD-1、TIGIT（T cell immuno receptor with immunoglobulin and ITIM domain）和杀伤细胞凝集素样受体G1（KLRG-1）的表达升高也与复发密切相关[37]。白血病干细胞上PD-L1的表达明显增高，使其能够抵御CTL的杀伤[41]。allo-HSCT后来自异体的免疫压力会增加T细胞抑制性受体的表达[3]，同时作为allo-HSCT后防治GVHD的方案，他克莫司也会增加PD-1的表达[42]。

（三）肿瘤微环境

除了改变肿瘤细胞本身以降低免疫原性、减少免疫攻击外，肿瘤细胞还可以通过调节微环境来加速疾病进展。大多数肿瘤细胞与微环境之间的研究聚焦于实体瘤中，最近在血液系统恶性肿瘤中也开始逐渐得到重视。

1. 细胞因子：血液肿瘤改变其周围免疫微环境的方式之一是增加抗炎细胞因子的产生从而阻碍免疫反应。以非移植背景为例，慢性髓性白血病（CML）细胞能产生转化生长因子-β（TGF-β），TGF-β能阻断IFN-γ诱导的MHC Ⅱ类基因的转录，从而下调MHC II类分子来降低CML细胞的免疫原性[43]。TGF-β通过使叉头转录因子（Forkhead O transcription factors, FoxO）停留在核内来增加Akt信号，Akt信号可以促进CML细胞增殖并阻碍其凋亡[44]。与TGF-β相似，IL-4也可降低MHC Ⅱ的表达以降低免疫原性[45]。

在向恶性细胞转化的过程中，髓系祖细胞会减少粒细胞集落刺激因子（G-CSF）、IL-15和IFN-γ的产生。在生理情况下，IL-15由正常髓系祖细胞产生，其生理功能是扩大和激活效应T细胞和NK细胞，并促进记忆干T细胞亚群的产生[46]。因此，微环境中IL-15浓度低更有利于白血病细胞生存。即使是不同的血液系统恶性肿瘤，血液中低水平IL-15都与移植后复发风险正相关[47]。此外，在allo-HSCT后特异性HLA丢失的复发患者中，FMS样酪氨酸激酶3基因内部串联重复序列（FLT3-ITD）突变者较多[22]，且突变的FLT3-ITD是AML细胞产生IL-15减少的原因之一[48]，这提示白血病细胞可能会通过某些特殊的机制以达到降低免疫原性和增加侵袭性的“一箭双雕”效果。

2. 免疫抑制细胞：间充质干细胞（MSC）是多能基质细胞，可分化成为成骨细胞、脂肪细胞等多种细胞类型；在生理条件下，MSC在成人中属于正常干细胞群存在于包括骨髓的各种组织中；因为MSC具有多系分化的能力，并能向全身各处趋化，因此也常常为肿瘤所用，包括协助实体瘤转移、生成新生血管、促进肿瘤增殖并抑制其凋亡，还能抑制免疫反应[49]。MSC与肿瘤微环境的具体相互作用仍不明确，许多研究都发现MSC既可以抑制肿瘤细胞增殖也可以抑制其凋亡[50]，特别是在allo-HSCT背景下由于供者免疫系统的加入会使其中千丝万缕的联系更加复杂。在小鼠allo-HSCT模型中，同时注射MSC与淋巴瘤细胞小鼠的淋巴瘤发生率低、CD8^+^T细胞比例增高，且同时改善了aGVHD[51]。在体外，MSC能使肿瘤细胞暂时停止在细胞周期的G_1_期；然而，当肿瘤细胞与MSC一起被注射到小鼠体内时，肿瘤的生长速度明显加快[52]。在一项前瞻性研究中，一组患者仅接受造血干细胞，而另一组接受造血干细胞与MSC共同移植，结果发现共同移植组GVHD发生率低，但复发率更高（60.0％对20.0％）[53]。

髓源抑制性细胞（MDSC）是肿瘤微环境的主要组成部分之一，他们自骨髓产生，可迁移到肿瘤中发挥强大的免疫抑制功能，生理情况下外周血中无法检出MDSC，但恶性肿瘤患者外周血中循环的MDSC数量增加[54]。MDSC可产生IL-10等免疫抑制性细胞因子，产生活性氧来消耗T细胞增殖所必须的氨基酸，还可通过自身CD40促进调节性T细胞（Treg）产生并诱导T细胞的免疫耐受，通过产生TGF-β来抑制NK细胞活性[55]。对于MSDC的研究主要集中于实体瘤，在血液系统肿瘤尚无MDSC的标准和定义，故确定参与免疫效应的MDSC亚型十分困难。目前只有一篇研究报道了存在CD1d表达的单核MDSC可以作为确认强抑制性亚型的标志[56]。在血液系统恶性肿瘤中，MDSC往往与不良预后相关[57]。在移植后30 d内，单核MDSC比例较高患者的复发概率明显高于其他患者[58]–[59]。但是一项研究发现，MDSC的出现并不意味着GVL效应下降：在allo-HSCT后的100 d内，外周血中MDSC的数量与严重aGVHD的发生有关，但与复发风险的增加无关[60]。这项观察结果表明，MDSC的数量并不一定与其功能正相关，即MDSC可能不会抑制GVL效应。但是目前在血液系统中MDSC的亚型尚未明确，故不同亚型的MDSC可能具有不同的功能，抑制活性强的MDSC亚型或许与allo-HSCT后复发相关。

Treg在allo-HSCT中的作用具有争议。移植前或移植后输注Treg细胞，在减少GVHD发生的同时，不会增加复发风险，反而可降低移植后复发率[61]–[62]。与普通的供者淋巴细胞输注（DLI）相比，输注去Treg供者淋巴细胞的患者具有更高的反应率、更长的无事件生存期，提示诱导GVL的同时不会出现过度的GVHD[63]。在肿瘤微环境中，Treg细胞具有免疫抑制性和抗凋亡性，有利于恶性造血细胞生存[64]。在小鼠模型中，Treg聚集于在白血病部位，阻碍了过继输注的CTL增殖及细胞毒作用[65]。当移除肿瘤微环境中的Treg后，局部CTL数量增加，病灶得以消退[64]–[65]。

二、allo-HSCT后复发的治疗策略

（一）过继性细胞输注

DLI主要利用CD4^+^ T细胞释放的细胞因子激活CTL而起到GVL效应，最终达到预防治移植后复发的目的。美国国家癌症研究所（NCI）推荐那些移植后复发且无GVHD的患者尝试DLI治疗[66]。在我国，目前尚缺乏allo-HSCT后复发患者使用DLI的大型回顾性及前瞻性研究数据资料。在北京大学人民医院等单位参与的多中心研究中，在血液学复发前预防性使用DLI患者的2年累积复发率低于未接受DLI的患者（46％对66％），3年总生存率（36％对11％）及无白血病生存率（29％对9％）也更高[67]。我中心小型回顾性研究发现，预防性使用DLI的患者1年总生存率为93.8％，而治疗性使用DLI的患者1年总生存率仅为27.3％[68]。

患者特异性HLA缺失不仅解释了白血病细胞如何逃避免疫监视，也部分解释了DLI的无效性——额外输注的供者T细胞无法找到攻击的靶点。一些研究认为，DLI的GVL效应与mHAg有关，即供者T细胞识别提呈的mHAg来启动杀伤，因为在DLI后患者外周血中可以检测到针对HA-1、HA-2的特异CTL，且CTL检出的时间与疾病缓解重合[69]。此外，对于那些与抑制性配体上调相关的复发人群，DLI与免疫检查点抑制剂联合可能会增强疗效。抗细胞毒性T淋巴细胞相关蛋白-4（CTLA-4）单抗与DLI联合使用不会加重GVHD，但会大幅提升GVL效应[70]。针对一些能被CTL识别的白血病抗原，如BCR-ABL、ETV6-AML1、蛋白酶-3、Wilms肿瘤基因（WT1），可选择性输注抗原特异性CTL[71]。然而，在HLA分子表达下调或缺失的情况下，此类治疗不能获益，因为免疫治疗的有效性在很大程度上取决于白血病细胞表达适量的HLA分子。

（二）二次移植

对于患者特异性HLA丢失复发人群，选择与丢失单链相合的供者可能是有效策略。临床研究发现，在HLA丢失复发后，选择更换供者的二次移植要比选择化疗等能获得更长的无病生存期[22]。尽管无HLA丢失的复发不需要通过更换供者来重新获得同种异体反应，但可能存在其他重获GVL效应的机制。

（三）嵌合抗原受体T细胞（CAR-T）治疗

在过去几年中，一些具有里程碑意义的细胞治疗利用基因编辑以非HLA限制的方式重新引导免疫细胞杀伤肿瘤细胞，其中最突出的例子是CAR-T细胞治疗。即嵌合抗原受体（CAR）来重新引导T细胞，该受体的胞外部分由一可与目标分子结合的特异性抗体组成，细胞内部分则是一个T细胞激活域[72]。CAR的细胞内信号域主要来自TCR/CD3复合物的CD3ζ链。T细胞的特异性抗体与肿瘤细胞表面的目标抗原结合后，信号分子中基于免疫酪氨酸受体激活基序（ITAM）被磷酸化并启动下游级联信号，最终诱导T细胞扩增、细胞因子分泌，最终导致肿瘤细胞溶解。

与依赖TCR激活T细胞不同，TCR对特定肽链-MHC的亲和度、MHC分子的数量、激活型共受体数量等都会影响T细胞对肿瘤细胞的杀伤效果[73]，CAR能够选择性与白血病细胞表面抗原结合，不依赖TCR-MHC相互作用，因此在HLA缺失或下调的复发患者中具有广阔的应用前景。除了对肿瘤靶抗原具有高亲和力外，CAR合成共刺激分子还不受肿瘤细胞或微环境所表达的抑制性免疫信号影响[74]。同时，CD8^+^T细胞亚群和CD4^+^ T细胞亚群都可以导入CAR，通过不依赖Fas或TNF-α信号通过颗粒酶/穿孔蛋白介导细胞溶解，且导入CAR的CD4^+^T细胞与CD8^+^T细胞一样，也可以对靶细胞进行细胞溶解作用[75]。

虽然CAR-T细胞通过非MHC限制途径识别靶抗原，但细胞需要识别在肿瘤细胞表面表达的抗原才能触发被激活，因此，若靶抗原在肿瘤细胞内表达则不能被CAR-T细胞识别，同时T细胞的激活可被针对CAR结合域的抗体阻断[76]。

（四）双特异性抗体

双特异性T细胞衔接系统（BiTE）可以使免疫细胞向白血病细胞重定向，作为“桥梁”使肿瘤抗原（如恶性B淋巴细胞的CD19或原始细胞的CD33）和免疫细胞（如T细胞上的CD3、NK细胞上的CD16）相互连接[77]。

倍林妥莫双抗（blinatumomab）是全球第一个上市的BiTE免疫治疗药物，能够同时连接肿瘤细胞的CD19和T细胞的CD3，用于治疗难治/复发急性B淋巴细胞白血病及淋巴瘤[78]。因其不依赖HLA行使功能，规避了TCR的HLA限制，从而使免疫突触形成。虽然它能快速且持久地清除骨髓中CD19^+^白血病细胞，但是倍林妥莫双抗对髓外病灶疗效差是其一大缺点：表现在对既往出现过髓外浸润病史或现存髓外病灶的患者治疗效果差；在开始治疗效果尚佳的患者，治疗后1/3以上都会出现髓外进展[79]。

除了已上市的BiTE产品，还有处在实验室阶段的新型BiTE。体外实验发现，抗CD3/CD33的双抗体使供者T细胞重新获得对HLA丢失白血病细胞的杀伤[80]。

（五）表观遗传学药物

临床上最常使用的两种去甲基药物（HMA）包括阿扎胞苷（Aza）和地西他滨（DAC），二者常用于AML和骨髓增生异常综合征（MDS）的治疗。HMA通过抑制DNA甲基转移酶改变了DNA的甲基化模式，诱导细胞周期停滞、细胞凋亡和分化；在造血干细胞移植的背景下，还能增强GVL效应[81]–[82]。一项纳入allo-HSCT后复发AML/MDS患者的研究发现，最能从Aza治疗获益的是那些在复发时白血病负荷较低（原始细胞<20％）的患者，且该研究还发现Aza与DLI联合使用并不能获益[83]。在小规模的病例报告中发现，在DAC治疗前仅为微小残留病（MRD）阳性的患者较血液学复发的患者更能获得长期MRD阴性完全缓解（CR）[84]。同时，HMA也是一把双刃剑，它可以上调PD-L1、PD-L2、PD-1和CTLA-4[85]，这可能与HMA使PD-1基因启动子去甲基化有关。PD-1基因启动子中CpG位点的去甲基化会导致T细胞表面的PD-1上调，特别是在被抑制功能的CD8^+^T细胞上常观察到PD-1基因启动子去甲基化[86]。这提示了在HMA治疗时，其治疗耐药或效果差与T细胞上PD-1上调有关，这也给HMA联合PD-1/PD-L1抗体治疗提供了依据。

组蛋白乙酰化改变在组蛋白乙酰转移酶（HAT）和组蛋白去乙酰化酶（HDAC）的活性之间保持平衡，是免疫调节的另一种表观遗传机制[87]。HDAC抑制剂（HDACi）伏立诺他（vorinostat）使组蛋白H3、H4乙酰化，下调血浆中TNF-α、IL-6等炎性细胞因子水平，增强Treg细胞的反应，但并不会降低效应T细胞对受者抗原的作用，还能保持NK细胞功能[87]。因此移植后存在GVHD的患者，在使用HDACi调节免疫的同时，还可保留GVL效应。在HDACi帕比司他（panobinostat）联合DLI预防AML/MDS患者allo-HSCT后复发的临床研究中，2年的复发率为20％，期间没有观察到严重的GVHD[88]。HDACi单独使用或与其他药物联合使用有望成为预防高危AML/MDS患者移植后复发的治疗策略。

（六）免疫检查点抑制剂

免疫检查点抑制剂（ICI）最先被用于实体瘤，PD-1和CLTA-4抑制剂彻底改变了对转移性黑色素瘤的治疗格局。PD-1通路作为检查点限制T细胞介导的免疫反应，PD-1的配体PD-L1和PD-L2都参与PD-1信号介导的T细胞免疫耐受，PD-L1^−/−^的小鼠模型中，移植野生型PD-L1基因的造血干细胞可引起严重的GVHD并快速死亡[89]。故在allo-HSCT背景下，PD-L1扮演维持受者免疫耐受的角色，抑制PD-1通路会增加患者GVHD的发生率及相关死亡率[90]；但上调的PD-L1会减弱供者T细胞的GVL效应[91]。因此，如何平衡GVHD和GVL效应对ICI的临床应用举足轻重。ICI使用的时机会影响GVHD和GVL效应间的平衡。小鼠模型中，移植后早期DLI会带来强烈的GVL效应，延迟DLI则无GVL效应。然而延迟DLI联合PD-L1单克隆抗体不仅可以带来GVL效应，还不会引起GVHD[92]；同样，延迟使用CTLA-4单抗在提高GVL效应的同时不会加重aGVHD[93]。当前，使用ICI治疗或预防allo-HSCT后复发的临床研究寥寥无几。在使用纳武利尤单抗（nivolumab，一种PD-1单抗）治疗allo-HSCT后复发的几项研究中，11例霍奇金淋巴瘤患者中10例对治疗有反应[94]–[96]。目前纳武利尤单抗或伊匹单抗（ipilimumab, CTLA-4单抗）治疗allo-HSCT复发后淋巴瘤患者的Ⅰ期临床试验正在进行（NCT01822509）；帕博利珠单抗（pembrolizumab, PD-1单抗）治疗移植后复发血液系统恶性肿瘤（淋系和髓系）随机对照试验也在多个中心进行中（NCT02981914、NCT03286114）。尽管移植后复发患者接受ICI治疗后缓解率较高，但同时也有较高的GVHD发生率[97]，这提示ICI在移植后患者的使用中具有潜在的免疫毒性。显然，需要大规模的前瞻性临床研究来获得allo-HSCT复发后使用ICI的安全性和有效性资料。

如前所述，HMA可上调PD-L1的表达，故将其与PD-L1/PD-1抑制剂联用或许可获得不错的效果。一项联合纳武利尤单抗和Aza治疗复发AML的Ⅱ期试验观察到了33％的总反应率[98]。肿瘤微环境中的细胞因子也会影响PD-L1/PD-1的表达，如IFN-γ能上调肿瘤细胞上PD-L1的表达水平[99]，故炎症细胞因子联合ICI有望在临床过程中得到应用。

三、总结与展望

allo-HSCT的格局正在迅速改变，随着治疗相关不良反应和死亡率的降低，人们对造血干细胞移植从“被动期待”转变为“主动出击”，从最初较盲目的“同一化”治疗转变对不同患者的“量体裁衣”。在这种新的情况下，探索移植后白血病细胞与免疫系统的相互作用至关重要。移植的成败很大程度在于能否充分利用免疫系统所赋予的诸多功能，并与靶向治疗相结合，尽可能多地击中恶性肿瘤细胞的弱点，减少选择性免疫逃逸的机会。新的肿瘤免疫靶点图谱定量系统、多组学单细胞技术、精度更高的人源化小鼠模型，都是造血干细胞移植免疫生物学知识版图扩张的驱动力。在临床上出现的“为什么”经由实验室技术推动而产生的新理论将成为开启问题答案的钥匙，并最终提高造血干细胞移植的疗效。
